# *EPHX1* and *GSTP1* polymorphisms are associated with COPD risk: a systematic review and meta-analysis

**DOI:** 10.3389/fgene.2023.1128985

**Published:** 2023-05-22

**Authors:** Qinjun Yang, Wanqiu Huang, Dandan Yin, Lu Zhang, Yating Gao, Jiabing Tong, Zegeng Li

**Affiliations:** ^1^ Anhui University of Chinese Medicine, Hefei, China; ^2^ Key Laboratory of Xin’An Medicine, Ministry of Education, Hefei, China; ^3^ The First Affiliated Hospital of Anhui University of Chinese Medicine, Hefei, China; ^4^ The First Affiliated Hospital of Anhui Medical University, Hefei, China; ^5^ Key Laboratory of Anhui Provincial Department of Education, Hefei, China

**Keywords:** COPD risk, EPHX1, GSTP1, gene polymorphism, meta-analysis

## Abstract

**Background:** Chronic obstructive pulmonary disease (COPD) affects approximately 400 million people worldwide and is associated with high mortality and morbidity. The effect of *EPHX1* and *GSTP1* gene polymorphisms on COPD risk has not been fully characterized.

**Objective:** To investigate the association of *EPHX1* and *GSTP1 gene* polymorphisms with COPD risk.

**Methods:** A systematic search was conducted on 9 databases to identify studies published in English and Chinese. The analysis was conducted following the Preferred Reporting Items for Systematic Reviews and Meta-Analyses reporting guidelines (PRISMA). The pooled OR and 95% CI were calculated to evaluate the association of *EPHX1* and *GSTP1 gene* polymorphisms with COPD risk. The I^2^ test, Q test, Egger’s test, and Begg’s test were conducted to determine the level of heterogeneity and publication bias of the included studies.

**Results:** In total, 857 articles were retrieved, among which 59 met the inclusion criteria. The *EPHX1* rs1051740 polymorphism (homozygote, heterozygote, dominant, recessives, and allele model) was significantly associated with high risk of COPD risk. Subgroup analysis revealed that the *EPHX1* rs1051740 polymorphism was significantly associated with COPD risk among Asians (homozygote, heterozygote, dominant, and allele model) and Caucasians (homozygote, dominant, recessives, and allele model). The *EPHX1* rs2234922 polymorphism (heterozygote, dominant, and allele model) was significantly associated with a low risk of COPD. Subgroup analysis showed that the *EPHX1* rs2234922 polymorphism (heterozygote, dominant, and allele model) was significantly associated with COPD risk among Asians. The *GSTP1* rs1695 polymorphism (homozygote and recessives model) was significantly associated with COPD risk. Subgroup analysis showed that the *GSTP1* rs1695 polymorphism (homozygote and recessives model) was significantly associated with COPD risk among Caucasians. The *GSTP1* rs1138272 polymorphism (heterozygote and dominant model) was significantly associated with COPD risk. Subgroup analysis suggested that the *GSTP1* rs1138272 polymorphism (heterozygote, dominant, and allele model) was significantly associated with COPD risk among Caucasians.

**Conclusion:** The C allele in *EPHX1* rs1051740 among Asians and the CC genotype among Caucasians may be risk factors for COPD. However, the GA genotype in *EPHX1* rs2234922 may be a protective factor against COPD in Asians. The GG genotype in *GSTP1* rs1695 and the TC genotype in *GSTP1* rs1138272 may be risk factors for COPD, especially among Caucasians.

## 1 Introduction

Chronic obstructive pulmonary disease (COPD) is a common disease that is characterized by persistent airflow limitation and the associated respiratory symptoms. Oxidative stress and chronic inflammation are important components of the mechanism contributing to COPD (Cheng et al., 2004; Global Initiative for Chronic Obstruc, 2021). COPD is the leading cause of lung disease-associated morbidity and mortality, and its incidence has been increasing globally (Singh et al., 2019). It has been predicted that by 2030, COPD will be the third leading cause of death worldwide, imposing a heavy socioeconomic burden (Nikolaou et al., 2020). COPD onset is closely correlated with airway and lung inflammation caused by harmful particles and smoke (Lareau et al., 2019; Global Initiative for Chronic Obstruc, 2021). However, only 10%–20% of chronic smokers exhibit COPD-associated severe lung dysfunction and the risk of airflow limitation varies greatly among smokers (Bascom, 1991; Pillai et al., 2009). Furthermore, patients with early-onset COPD exhibit familial aggregation (Silverman et al., 1998), indicating that COPD is a complex disease that is caused by interactions between genetic and environmental factors 9). Genome-wide association studies (GWAS) have reported many COPD-associated susceptibility genes (Sakornsakolpat et al., 2019; Shrine et al., 2019). Several studies have also shown that gene polymorphisms play a key role in COPD pathogenesis (Xiao et al., 2004; An et al., 2016a; Cho et al., 2022). Thus, the key genetic variations associated with COPD susceptibility need to be identified to improve COPD prevention and treatment.

Cigarette smoke contains many toxic constituents which stimulate the release of vast amounts of reactive oxygen species (ROS) and reactive nitrogen species (RNS) by airway epithelial cells, granulocytes, and macrophages, leading to oxidative stress, oxidative inactivation of antiproteases, alveolar epithelial damage, increased neutrophils in pulmonary microvessels, and enhanced proinflammatory gene expression. Some of the genes involved in the metabolism of toxic substances found in cigarette smoke are thought to participate in COPD pathogenesis (Joos et al., 2002; Silverman, 2020). For instance, glutathione S-transferase P1 (*GSTP1*) and microsomal epoxide hydrolase (*EPHX1*) are typical detoxification enzyme genes known to be highly expressed in the lungs and are closely associated with oxidative stress and inflammatory responses in COPD (Tomaki et al., 2007).

*EPHX1*, which is involved in the metabolism and detoxification of exogenous chemicals, plays a key role in general oxidative defense in the lungs (Sandford and Silverman, 2002). The *EPHX1* gene (≈35.48 kb long) is located on chromosome 1q42.1 and contains 9 exons and 8 introns (Akparova et al., 2017). Mutations of exon 3 Tyr113His (rs1051740) and exon 4 His139Arg (rs2234922) are the most common polymorphisms that influence the *EPHX1* enzyme activities (Kiyohara et al., 2006). Genetic correlation case-control studies have shown that *EPHX1* rs1051740 genetic variations increased the risk of COPD (Hersh et al., 2005; Hersh et al., 2006; Hersh et al., 2007), whereas *EPHX1* rs2234922 variation decrease the risk of COPD (Smith and Harrison, 1997). However, these observations are controversial because some studies did not find any correlations between these polymorphisms and COPD risk (Brøgger et al., 2006; Chappell et al., 2008). *GSTP1* (≈3 kb long), a member of the GST superfamily, is located on chromosome 11q13 and contains 6 introns and 7 exons. Compared to other GSTs, it is highly expressed in respiratory tissues including the alveoli, alveolar macrophages as well as bronchioles (Cantlay et al., 1994). *GSTP1* catalyzes various electrophiles and glutathione, and serves to eliminate the products in tobacco smoke that cause toxicity associated with electrophiles and oxidative stress (Rodriguez et al., 2005; Du et al., 2019). Exon 5 Ile105Val (rs1695) and exon 6 Ala114Val (rs1138272) are the main *GSTP1* polymorphisms (Cheng et al., 2004). Although studies have investigated the association of *EPHX1* and *GSTP1* gene polymorphisms with the risk of COPD in different ethnic groups, findings from such studies have been inconsistent which may be attributed to the small sample sizes in the studies. Here, we conducted a systematic review and meta-analysis to determine the association of *EPHX1* (rs1051740 and rs2234922) and *GSTP1* (rs1695 and rs1138272) polymorphisms with the risk of COPD risk, with the aim of providing evidence-based information on COPD pathogenesis which can be used to develop potential strategies for its diagnosis, prevention and treatment. The analysis was conducted in line with the PRISMA 2020 (Supplementary Table S1).

## 2 Materials and methods

### 2.1 Search strategy

A search was conducted on the PubMed, Embase, Web of Science, Cochrane Library, SCOPUS, CENTRAL, CINAHL, CNKI, and WANFANG DATA to identify relevant studies published up to 31 September 2022. Search terms included “COPD”, “gene”, “gene variation”, “single nucleotide polymorphism”, “*EPHX1*”, and “*GSTP1*”. Detailed retrieval strategies are provided in Supplementary Table S2. References in the retrieved literature were also reviewed.

### 2.2 Inclusion and exclusion criteria

The inclusion criteria for studies were: ①Explored the association of *EPHX1* rs1051740, rs2234922, and *GSTP1* rs1695, rs1138272 polymorphisms with the risk of COPD; ②case-control studies; ③ Reported specific genotype and allele counts or significant allele frequency (MAF) between groups; and ④ Involved human subjects.

The exclusion criteria for studies were: ① Were repeat published studies; ②Case reports, comments, or expert opinions; ③ Included other genetic polymorphisms; ④ Involved a non-healthy (with other diseases, such as lung cancer) control group; and ⑤ Lacked data that could be extracted from text, tables, or charts, or that could be obtained from the authors upon request.

### 2.3 Data extraction and literature quality evaluation

Two researchers (QY and WH) independently searched for the articles, assessed the inclusion/exclusion parameters, and conducted data extraction. Any discrepancies were resolved through discussions between two reviewers (JT and ZL). The extracted data included the first author’s name, year of publication, sample size, ethnicity, genotype, genotyping method, genotype count, allele count, and whether it met the Hardy Weinberger equilibrium (HWE). Using the Newcastle-Ottawa Scale (NOS) (Stang, 2010), the quality of included studies was evaluated based on the following criteria: selection of research subjects, comparability between groups, and outcome measurements. Studies with NOS scores ≥6 were considered high-quality studies (Mirzakhani et al., 2020).

### 2.4 Statistical analysis

The association of *EPHX1* (rs1051740 and rs2234922) and *GSTP1* (rs1695 and rs1138272) polymorphisms with the risk of COPD was analyzed using homozygote, heterozygote, dominant, recessive, and allele models. Higgin’s I^2^ and Cochran’s Q tests were used to evaluate heterogeneity between studies. Where heterogeneity was significant (I^2^ >50%, *P*_Q_<0.10), the random-effects model (DerSimonian–Lloyd method) was used, and in contrast (I^2^ ≤ 50%, *P*_Q_≥0.10), the fixed-effects model (Mantel‒Haenszel method) was used. The pooled odds ratio (OR) and 95% confidence interval (95% CI) were used as effect measurement indicators for each result. Furthermore, we conducted subgroup analyses according to different ethnic information. Egger’s and Begg’s tests were used to assess the publication bias, with *p* > 0.05 indicating no significant publication bias. If publication bias was found, the trim-and-fill method was used to assess the stability of the pooled results, and a funnel plot was drawn. A non-significant change in *p* values indicated that publication bias had little influence on the results. Sensitivity analysis was performed by excluding the studies one by one to determine the impact of each study on the total effects value and to assess the stability of results. All statistical analyses were done using Stata 17.0 (Stata Corp, College Station, TX, United States).

## 3 Results

### 3.1 Document screening process and results

**FIGURE 1:**
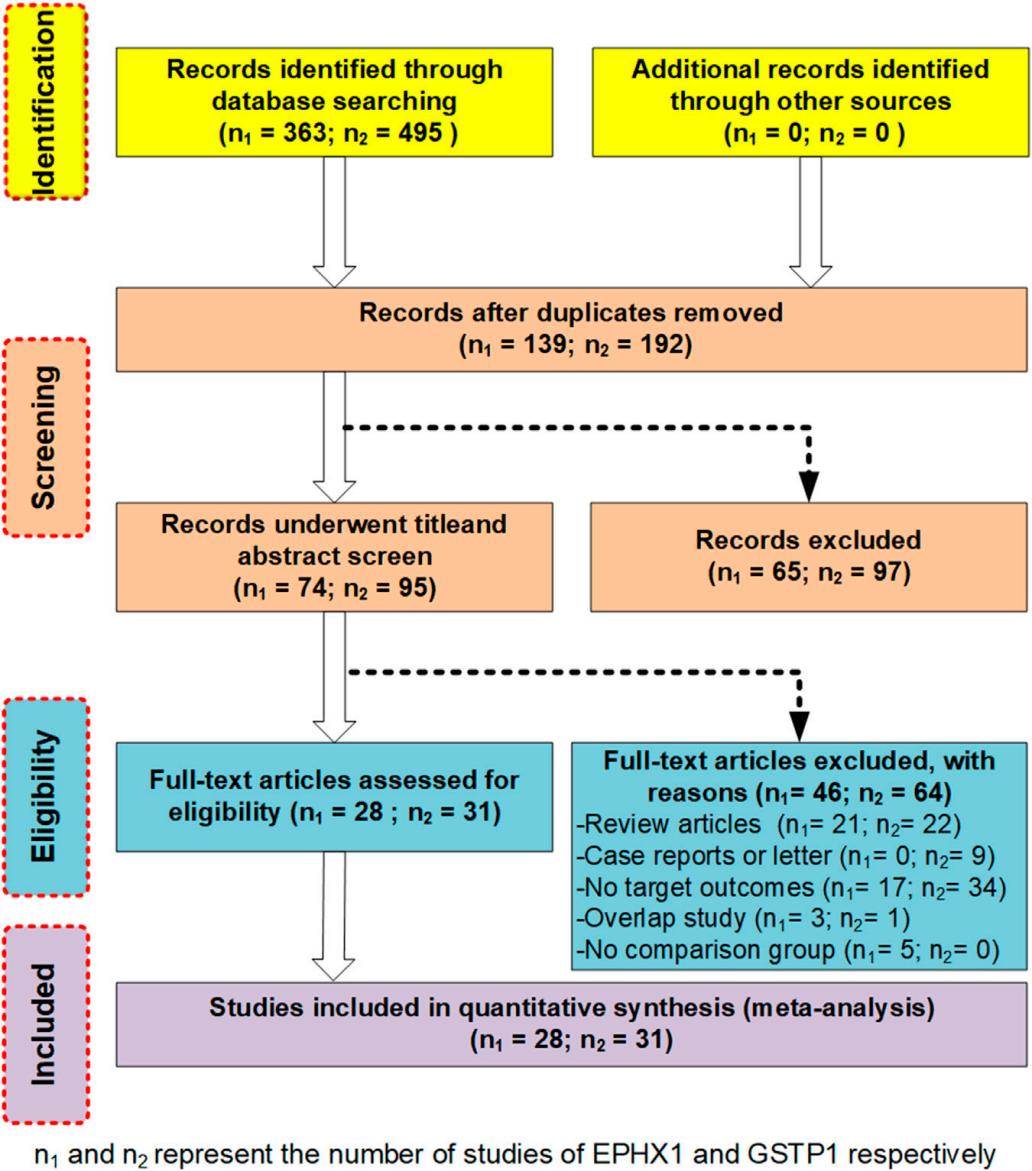
Schematic presentation of the literature screening process.

We employed an unrestricted literature search and a repeated search review procedure. Based on our inclusion and exclusion criteria, 59 articles containing genetic data were selected for analysis. Among them, 28 investigated *EPHX1* rs1051740 and involved 5007 cases and 5476 controls, 26 explored *EPHX1* rs2234922 and involved 4840 cases and 5326 controls, 31 investigated *GSTP1* rs1695 and comprised 3975 cases and 4301 controls, while 7 explored *GSTP1* rs1138272 and involved 1170 cases and 1455 controls. A schematic presentation of the literature screening process is shown in Figure 1. Based on the NOS scoring analysis of case controls, 16 studies had NOS scores of 6, while 43 had NOS scores of≥7, indicating that the included studies were of high quality. The basic characteristics of the included studies including the authors, publication years, NOS scores, ethnicity, genotype distributions of the case and control groups, and Hardy–Weinberg equilibrium analysis of the control group gene distribution are shown in Table 1 and Table 2.

**TABLE 1:** Essential characteristics of *EPHX1* (rs1051740 and rs2234922) polymorphism in the included studies

First author	Year	NOS score	Ethnicity	*EPHX1* rs1051740											*EPHX1* rs2234922										
				Case					Control					*P*_HWE_	Case					Control					*P*_HWE_
				CC	CT	TT	C	T	CC	CT	TT	C	T		GG	GA	AA	G	A	GG	GA	AA	G	A	
Smith and Harrison (1997)	1997	7	Caucasian	13	28	27	54	82	13	99	91	125	281	0.121	2	29	37	33	103	3	53	147	59	347	0.768
Yim et al. (2000)	2000	7	Asian	36	23	24	95	71	36	23	24	95	71	0.000	8	16	59	32	134	2	17	57	21	131	0.869
Rodriguez et al. (2002)	2002	6	Caucasian	8	32	39	48	110	2	58	86	62	230	0.076	0	32	47	32	126	4	48	94	56	236	0.765
Zhang et al. (2002)	2002	6	Asian	25	15	15	65	45	20	20	12	60	44	0.310	5	10	40	20	90	4	12	36	20	84	0.179
Zhang (2002)	2002	7	Asian	25	15	15	65	45	20	20	12	60	44	0.311	5	10	40	20	90	4	12	36	20	84	0.179
Park et al. (2003)	2003	6	Asian	8	32	18	48	68	27	24	27	78	78	0.003	1	13	43	15	99	3	20	55	26	130	0.794
Korytina et al. (2003)	2003	7	Caucasian	5	43	40	53	123	1	60	101	62	262	0.043	1	22	68	24	158	5	32	127	42	286	0.271
Cheng et al. (2004)	2004	6	Asian	67	84	33	218	150	64	92	56	220	204	0.163	3	43	138	49	319	7	66	139	80	344	0.97
Xiao et al. (2004)	2004	8	Asian	38	42	20	118	82	39	32	29	110	90	0.001	0	17	83	17	183	0	18	82	18	182	0.613
Hersh et al. (2005)	2005	7	Caucasian	26	125	153	177	431	35	177	229	247	635	0.995	9	86	209	104	504	21	152	268	194	688	0.996
Park et al. (2005)	2005	8	Caucasian	19	45	67	83	179	17	92	153	126	398	0.822	4	44	82	52	208	16	93	153	125	399	0.934
Brøgger et al. (2006)	2006	7	Caucasian	12	117	110	141	337	26	94	121	146	336	0.495	10	83	145	103	373	12	86	150	110	386	0.997
Matheson et al. (2006)	2006	7	Caucasian	9	25	38	43	101	24	95	101	143	297	0.973	5	21	46	31	113	7	74	139	88	352	0.750
Fu et al. (2007)	2007	6	Asian	45	157	54	247	265	48	100	118	196	336	0.007	12	10	204	34	418	12	49	205	73	459	0.001
Vibhuti et al. (2007)	2007	6	Asian	75	75	52	225	179	31	51	54	113	159	0.029	10	59	133	79	325	8	56	72	72	200	0.767
Chappell et al. (2008)	2008	8	Caucasian	92	417	508	601	1433	91	374	447	556	1268	0.620	41	336	640	418	1616	46	283	583	375	1449	0.319
Zidzik et al. (2008)	2008	8	Caucasian	42	70	105	154	280	15	67	78	97	223	0.994	8	69	140	85	349	6	63	91	75	245	0.47
Zheng and Zheng (2009)	2009	6	Asian	36	22	22	94	66	34	33	20	101	73	0.119	7	15	58	29	131	7	20	60	34	140	0.043
Penyige et al. (2010)	2010	7	Caucasian	19	122	127	160	376	25	110	154	160	418	0.703	8	92	169	108	430	12	102	171	126	444	0.803
Lakhdar et al. (2010a)	2010	8	Caucasian	36	96	102	168	300	14	82	83	110	248	0.596	6	86	142	98	370	4	59	119	67	297	0.565
Chen et al. (2011)	2011	7	Asian	61	28	16	150	60	36	43	24	115	91	0.297	—	—	—	—	—	—	—	—	—	—	—
Zhang et al. (2015)	2015	8	Asian	38	140	41	216	222	46	85	92	177	269	0.010	10	36	173	56	382	8	45	170	61	385	0.095
El Wahsh et al. (2015)	2015	6	Caucasian	10	36	100	56	236	6	22	102	34	226	0.014	12	34	100	58	234	2	50	78	54	206	0.157
Stanković et al. (2015)	2015	7	Caucasian	16	48	58	80	164	12	40	48	64	136	0.721	2	38	82	42	202	5	29	66	39	161	0.748
Akparova et al. (2017)	2017	8	Asian	8	23	24	39	71	3	12	37	18	86	0.377	—	—	—	—	—	—	—	—	—	—	—
Zhang and Xu (2018)	2018	8	Asian	10	28	28	48	84	5	20	60	30	140	0.214	4	23	38	31	99	5	31	49	41	129	0.999
An et al. (2019a)	2019	8	Asian	52	149	109	253	367	41	102	61	184	224	0.99	8	84	218	100	520	6	56	141	68	338	0.988
Ganbold et al. (2021)	2021	8	Asian	24	61	96	109	253	31	103	158	165	419	0.085	8	55	118	71	291	16	103	173	135	449	0.992

**TABLE 2:** Essential characteristics of *GSTP1* (rs1695 and rs1138272) polymorphism in the included studies.

First author	Year	NOS score	Ethnicity	*GSTP1* rs1695											*GSTP1* rs1138272										
				Case					Control					*P*_HWE_	Case					Control					*P*_HWE_
				GG	GA	AA	G	A	GG	GA	AA	G	A		TT	TC	CC	T	C	TT	TC	CC	T	C	
Harries et al. (1997)	1997	6	Caucasian	10	35	34	55	103	10	66	79	86	224	0.742	—	—	—	—	—	—	—	—	—	—	—
Ishii et al. (1999)	1999	8	Asian	0	11	42	11	95	2	22	26	26	74	0.598	0	0	53	0	106	0	0	50	0	100	—
Yim et al. (2002)	2002	7	Asian	2	24	63	28	150	2	35	57	39	149	0.439	—	—	—	—	—	—	—	—	—	—	—
Lu and He (2002)	2002	6	Asian	5	22	70	32	162	2	24	41	28	106	0.791	—	—	—	—	—	—	—	—	—	—	—
Xiao et al. (2004)	2004	8	Asian	1	29	70	31	169	3	40	57	46	154	0.433	—	—	—	—	—	—	—	—	—	—	—
Zhang et al. (2003)	2003	7	Asian	5	5	37	15	79	1	3	44	5	91	0.039	—	—	—	—	—	—	—	—	—	—	—
Gaspar et al. (2004)	2004	7	Caucasian	5	35	35	45	105	7	36	47	50	130	1.000	—	—	—	—	—	—	—	—	—	—	—
Cheng et al. (2004)	2004	7	Asian	9	78	97	96	272	15	98	99	128	296	0.371	—	—	—	—	—	—	—	—	—	—	—
Korytina et al. (2004)	2004	7	Caucasian	7	35	62	49	159	5	55	104	65	263	0.778	—	—	—	—	—	—	—	—	—	—	—
Ma et al. (2004)	2004	6	Asian	7	32	65	46	162	3	18	23	24	64	0.979	—	—	—	—	—	—	—	—	—	—	—
Rodriguez et al. (2005)	2005	7	Caucasian	10	36	52	56	140	13	88	97	114	282	0.497	—	—	—	—	—	—	—	—	—	—	—
Hu et al. (2005)	2005	7	Asian	2	3	45	7	93	4	5	59	13	123	1.326	—	—	—	—	—	—	—	—	—	—	—
Li (2005)	2005	8	Asian	1	16	74	18	164	4	18	65	26	148	0.222	—	—	—	—	—	—	—	—	—	—	—
Calikoglu et al. (2006)	2006	7	Asian	14	42	88	70	218	36	57	57	129	171	0.023	—	—	—	—	—	—	—	—	—	—	—
Fang et al. (2006)	2006	7	Asian	1	16	74	18	164	4	18	65	26	148	0.222	—	—	—	—	—	—	—	—	—	—	—
Vibhuti et al. (2007)	2007	6	Caucasian	22	75	105	119	285	4	42	90	50	222	0.944	22	57	123	101	303	6	24	106	36	236	0.026
Chan-Yeung et al. (2007)	2007	8	Asian	8	43	112	59	267	2	47	112	51	271	0.484	—	—	—	—	—	—	—	—	—	—	—
Korytina et al. (2009)	2009	7	Caucasian	13	86	217	112	520	14	232	329	260	890	0.001	5	72	236	82	544	10	79	426	99	931	0.029
Lakhdar et al. (2010b)	2010	7	Caucasian	49	104	81	202	266	19	79	84	117	247	0.998	0	0	234	0	468	0	0	182	0	364	—
Khan et al. (2012)	2012	6	Asian	34	58	94	126	246	13	56	91	82	238	0.586	—	—	—	—	—	—	—	—	—	—	—
Ling et al. (2013)	2013	6	Asian	14	33	103	61	239	10	22	118	42	258	1.053	—	—	—	—	—	—	—	—	—	—	—
Zuntar et al. (2014)	2014	6	Caucasian	4	16	10	24	36	1	25	34	27	93	0.321	7	21	2	35	25	10	25	25	45	75	0.690
Wu et al. (2014)	2014	8	Asian	19	18	113	56	244	7	11	132	25	275	1.558	—	—	—	—	—	—	—	—	—	—	—
Stanković et al. (2015)	2015	7	Caucasian	15	59	48	89	155	9	46	45	64	136	0.850	—	—	—	—	—	—	—	—	—	—	—
El-Gazzar et al. (2016)	2016	7	Caucasian	13	17	0	43	17	12	8	0	32	8	0.535	—	—	—	—	—	—	—	—	—	—	—
He et al. (2016)	2016	8	Asian	1	14	47	16	108	1	21	40	23	101	0.635	—	—	—	—	—	—	—	—	—	—	—
Du et al. (2019)	2019	6	Asian	33	72	45	138	162	22	61	67	105	195	0.433	3	96	18	102	132	26	82	42	134	166	0.431
An et al. (2019b)	2019	6	Asian	7	12	31	26	74	4	6	40	14	86	0.002	—	—	—	—	—	—	—	—	—	—	—
An et al. (2019a)	2019	8	Asian	17	110	183	144	476	12	76	115	100	306	0.993	—	—	—	—	—	—	—	—	—	—	—
Chen (2020)	2020	8	Asian	7	70	147	84	364	4	58	162	66	382	0.900	—	—	—	—	—	—	—	—	—	—	—
Ganbold et al. (2021)	2021	8	Asian	20	81	80	121	241	26	122	144	174	410	1.000	14	74	93	102	260	27	124	141	178	406	1.000

### 3.3 Association between the *EPHX1* rs1051740 polymorphism and COPD risk

The results of meta-analyses of various models and subgroup analyses were utilized to explore the association of *EPHX1* rs1051740 polymorphism with the COPD risk as shown in Table 3. In the overall analysis, heterogeneity among the five genetic models was high (I^2^>50%, *P*_Q_<0.10). Therefore, the random-effects model was used to determine the pooled OR and its 95% CI. For the homozygote model (OR = 1.460, *P*_Z_ = 0.001), heterozygote model (OR = 1.275, *P*_Z_ = 0.007), dominant model (OR = 1.340, *P*_Z_ = 0.000), recessive model (OR = 1.296, *P*_Z_ = 0.011) and allele model (OR = 1.254, *P*_Z_ = 0.000), *EPHX1* rs1051740 was significantly associated with COPD risk, indicating that the C allele is a risk factor for COPD. Subgroup analysis based on ethnicity showed that in the homozygote model (OR = 1.453, *P*_Z_ = 0.007), heterozygote model (OR = 1.465, *P*_Z_ = 0.027), dominant model (OR = 1.520, *P*_Z_ = 0.005), and allele model (OR = 1.308, *P*_Z_ = 0.002), *EPHX1* rs1051740 was significantly associated with COPD risk among Asians, suggesting that the C allele is a risk factor for COPD in Asians. In addition, we also found that in the homozygote model (OR = 1.497, *P*_Z_ = 0.025), dominant model (OR = 1.14, *P*_Z_ = 0.028), recessive model (OR = 1.482, *P*_Z_ = 0.033) and allele model (OR = 1.186, *P*_Z_ = 0.005), *EPHX1* rs1051740 was significantly associated with COPD risk among Caucasians, indicating that the CC genotype is a risk factor for COPD in Caucasians. Since 8 studies had *P*_HWE_<0.05 (Yim et al., 2000; Korytina et al., 2003; Park et al., 2003; Xiao et al., 2003; Xiao et al., 2004; Fu et al., 2007; Vibhuti et al., 2007; El Wahsh et al., 2015; Zhang et al., 2015), we conducted a subgroup analysis involving 20 studies with *P*_HWE_≥0.05. It was found that *EPHX1* rs1051740 [homozygote model (OR = 1.409, 95% CI = 1.093–1.817, *P*_Z_ = 0.008), recessive model (OR = 1.411, 95% CI = 1.116–1.784, *P*_Z_ = 0.004) and allele model (OR = 1.203, 95% CI = 1.071–1.352, *P*_Z_ = 0.002)] was significantly associated with COPD risk, consistent with findings from the overall analysis. Thus, studies with *P*_HWE_<0.05 did not affect the overall results, and the findings were generally reliable.

**TABLE 3:** Meta-analysis results of the association between *EPHX1* rs1051740 and COPD risk.

Genetic models	Ethnicity	Studies	Association test		M	Heterogeneity test		Publication bias	
			OR (95% CI)	*P*_Z_		I^2^ (%)	*P*_Q_	Egger’s test	Bgge’s test
**Homozygote model CC vs. TT**	Overall	28	1.460 (1.177–1.811)	0.001	R	59.80	0.000	0.031	0.114
	Caucasian	13	1.497 (1.053–2.128)	0.025		66.00	0.000		
	Asian	15	1.453 (1.105–1.910)	0.007		53.90	0.007		
**Heterozygote model CT vs. TT**	Overall	28	1.275 (1.069–1.521)	0.007	R	71.20	0.000	0.397	0.502
	Caucasian	13	1.091 (0.962–1.238)	0.174		18.70	0.255		
	Asian	15	1.465 (1.004–2.055)	0.027		78.00	0.000		
**Dominant model CC + CT vs. TT**	Overall	28	1.340 (1.147–1.566)	0.000	R	68.00	0.000	0.099	0.477
	Caucasian	13	1.140 (1.015–1.280)	0.028		15.80	0.285		
	Asian	15	1.520 (1.138–2.031)	0.005		74.90	0.000		
**Recessive model CC vs. CT + TT**	Overall	28	1.296 (1.061–1.583)	0.011	R	62.80	0.000	0.029	0.058
	Caucasian	13	1.482 (1.033–2.125)	0.033		70.00	0.000		
	Asian	15	1.197 (0.944–1.519)	0.139		56.70	0.004		
**Allele model C vs. T**	Overall	28	1.254 (1.128–1.393)	0.000	R	64.10	0.000	0.023	0.179
	Caucasian	13	1.186 (1.053–1.335)	0.005		48.50	0.025		
	Asian	15	1.308 (1.100–1.555)	0.002		69.60	0.000		

Notes: M: model; R: random-effects model.

**FIGURE 2:**
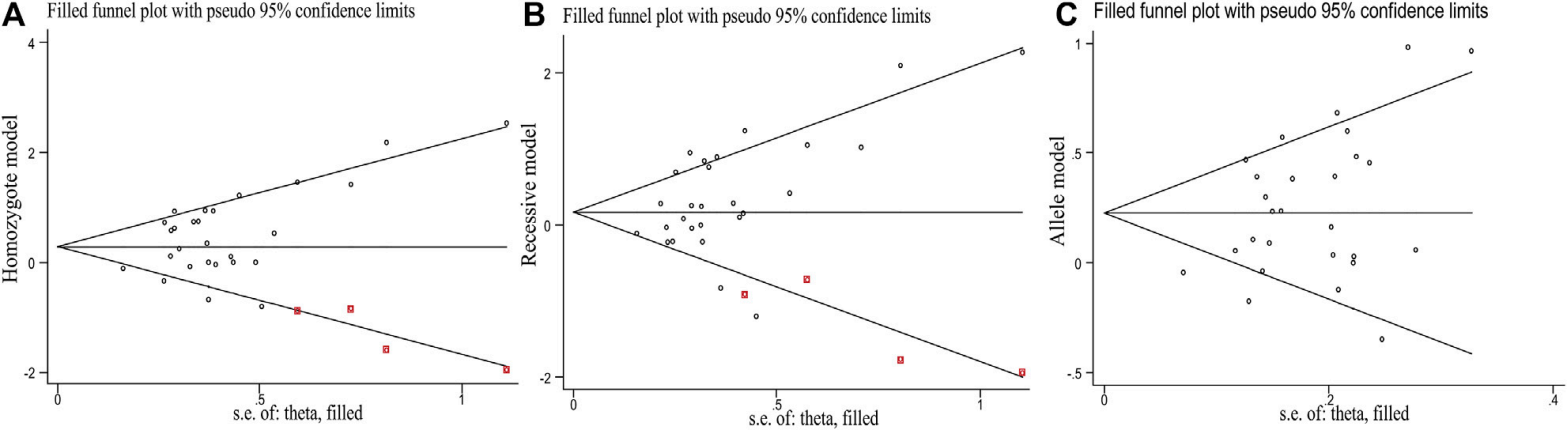
Funnel diagram of the trim-and-fill method of the EPHX1 rs1051740 polymorphism (Red points represent the supplementary studies). **(A)** Homozygote model. **(B)** Reccssive model. **(C)** Allele model.

**FIGURE 3:**
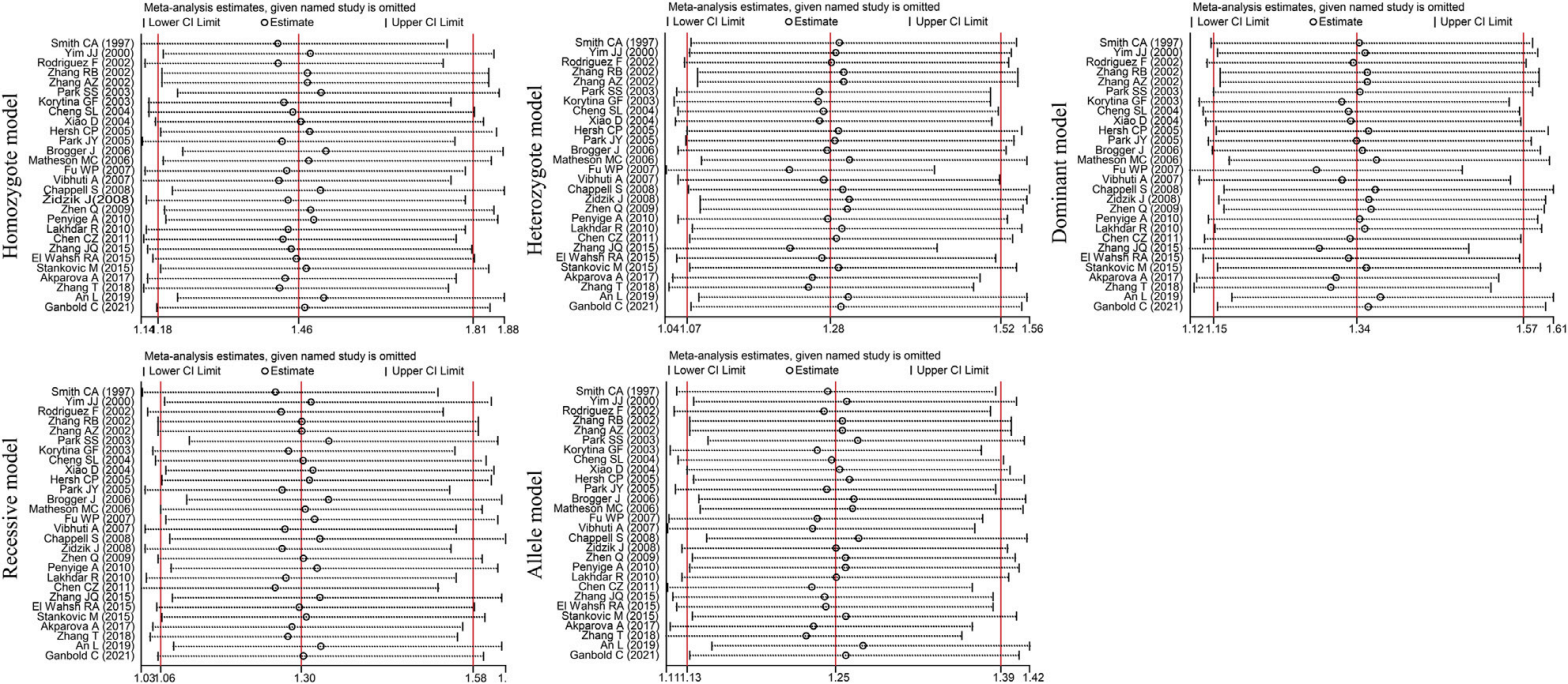
Sensitivity analysis diagram of the *EPHX1* rs1051740 polymorphism.

Egger’s test [homozygote model (*P*_Egger_ = 0.031), recessive model (*P*_Egger_ = 0.029) and allele model (*P*_Egger_ = 0.023)] showed that publication bias might have been present, but Begg’s test did not reveal any publication bias (*P*_Bgge_>0.05) (*Table 3*). The nonparametric trim-and-fill method was used to reduce the deviation in the combined effects. The trim-and-fill method is used to correct the impact of publication bias on the combined effect of meta-analysis. If the result of funnel plots show symmetry and *P*_*z*_ > 0.05 based on the trim-and-fill method results, the publication bias is not considered to significantly affect the reliability of the pooled results (Luo et al., 2022). We found that there were no significant changes in amounts of effects before and after pruning in the homozygote model (4 supplementary studies, OR = 1.331, 95% CI = 1.065–1.664, *P*_Z_ = 0.012) and the allele model (3 excluded studies, OR = 1.254, 95% CI = 1.128–1.393, *P*_Z_ = 0.000), indicating that the publication bias was too small to affect the stability of subgroup analysis results (Figures 2A, C). However, the shapes of the funnel plots were not symmetrical in the recessive models and *P*z>0.05, indicating that the pooled results of the recessive model might not be stable (4 supplementary studies, OR = 1.254, 95% CI = 1.128–1.393, *P*_Z_ = 0.567) (*Figure 2B*). Given the high heterogeneity (I^2^>50%, *P*_Q_<0.05) among the five genetic models, we conducted sensitivity analyses by excluding the studies one by one. The OR values of all studies fell within the 95% CI (Figure 3), which indicated that the overall estimates were stable and the results were reliable.

### 3.4 Association between *EPHX1* rs2234922 polymorphism and COPD risk

The results of meta-analyses of various models and the subgroups used to explore the association between *EPHX1* rs2234922 polymorphism and COPD risk are summarized in Table 4 *.* In the overall analysis, heterogeneity among the five genetic models was low (I^2^<50%, *P*_Q_>0.10), and thus the fixed-effects model was used to determine the pooled OR and it is 95% CI. For the heterozygote model (OR = 0.885, *P*_Z_ = 0.007), dominant model (OR = 0.886, *P*_Z_ = 0.005) and allele model (OR = 0.906, *P*_Z_ = 0.007), *EPHX1* rs2234922 was found to be significantly associated with a lower risk of COPD, indicating that the G allele may be a protective factor for COPD. Subgroup analyses based on ethnicity showed that in the heterozygote model (OR = 0.716, *P*_Z_ = 0.000), dominant model (OR = 0.752, *P*_Z_ = 0.000) and allele model (OR = 0.817, *P*_Z_ = 0.002), *EPHX1* rs2234922 was significantly associated with reduced COPD risk among Asians, indicating that the GA genotype may be a protective factor for COPD among Asians. Considering that 2 studies had *P*_HWE_<0.05 (Fu et al., 2007; Zheng and Zheng, 2009), we conducted a subgroup analysis of 24 studies with *P*_HWE_≥0.05. Results showed that *EPHX1* rs2234922 [dominant model (OR = 0.912, 95% CI = 0.836–0.994, *P*_Z_ = 0.037) and allele model (OR = 0.922, 95% CI = 0.857–0.933, *P*_Z_ = 0.032)] were associated with a lower risk of COPD, consistent with findings from the overall analysis, and indicating that some studies with *P*_HWE_ <0.05 did not affect the overall results, which were reliable.

**TABLE 4:** Meta-analysis results of the association between *EPHX1* rs2234922 and COPD risk.

Genetic models	Ethnicity	Studies	Association test		M	Heterogeneity test		Publication bias	
			OR (95% CI)	*P*_Z_		I^2^ (%)	*P*_Q_	Egger’s test	Begg’s test
**GG vs. AA Homozygote model**	Overall	25	0.873 (0.713–1.070)	0.190	F	0.00	0.673	0.555	0.513
	Caucasian	13	0.828 (0.637–1.076)	0.157		15.00	0.294		
	Asian	12	0.947 (0.686–1.306)	0.738		0.00	0.874		
**GA vs. AA Heterozygote model**	Overall	26	0.885 (0.810–0.967)	0.007	F	48.70	0.003	0.241	0.366
	Caucasian	13	0.978 (0.878–1.089)	0.684		46.90	0.031		
	Asian	13	0.716 (0.612–0.838)	0.000		30.90	0.136		
**GG + GA vs. AA Dominant model**	Overall	26	0.886 (0.814–0.964)	0.005	F	38.20	0.026	0.663	0.774
	Caucasian	13	0.962 (0.867–1.067)	0.461		38.50	0.077		
	Asian	13	0.752 (0.814–0.964)	0.000		15.00	0.293		
**GG vs. GA + AA Recessive model**	Overall	25	0.915 (0.748–1.118)	0.383	F	0.00	0.633	0.526	0.484
	Caucasian	13	0.844 (0.652–1.094)	0.200		18.70	0.255		
	Asian	12	1.033 (0.751–1.422)	0.840		0.00	0.905		
**G vs. A Allele model**	Overall	26	0.906 (0.843–0.973)	0.007	F	24.80	0.150	0.899	0.523
	Caucasian	13	0.953 (0.873–1.041)	0.285		26.80	0.174		
	Asian	13	0.817 (0.720–0.926)	0.002		7.60	0.370		

Notes: M, model; F, fixed-effects model.

**FIGURE 4:**
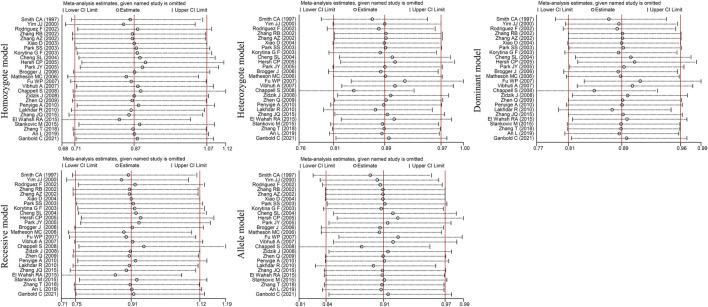
Sensitivity analysis diagram of the *EPHX1* rs2234922 polymorphism.

Egger’s and Begg’s tests of the included studies did not reveal any publication bias (*P*_Egger_>0.05, *P*_Bgge_>0.05) (*Table 4)*. Sensitivity analysis was conducted by excluding the studies one by one. The OR values of all studies fell within the 95% CI (Figure 4). These results suggested that the overall estimates were stable and the results were reliable.

### 3.5 Association between the *GSTP1* rs1695 polymorphism and COPD risk

The results of the meta-analysis among various models and subgroups used to explore the association between *GSTP1* rs1695 and COPD risk are summarized in Table 5. In the overall analysis, the heterogeneity of the recessive model was low (I^2^ = 45.40%, *P*_Q_ = 0.004), and therefore, the fixed-effects model was used to determine the pooled OR and its 95% CI. The other models had significant heterogeneity (I^2^>50%, *P*_Q_<0.10). Thus, the random-effects model was adopted. For the homozygote model (OR = 1.434, *P*_Z_ = 0.022) and recessive model (OR = 1.395, *P*_Z_ = 0.000), *GSTP1* rs2234922 was found to be significantly associated with COPD risk, indicating that the GG genotype is a risk factor for COPD. Subgroup analysis based on ethnicity showed that in the homozygote model (OR = 2.064, *P*_Z_ = 0.000) and recessive model (OR = 1.850, *P*_Z_ = 0.000), *GSTP1* rs1695 was significantly associated with COPD risk among Caucasians, while the association among Asians was not significant. Since 4 studies had *P*_HWE_ <0.05 (Zhang et al., 2003; Calikoglu et al., 2006; Korytina et al., 2009; An et al., 2019b), we conducted a subgroup analysis of 27 studies with *P*_HWE_≥0.05 and established that *GSTP1* rs1695 [homozygote model (OR = 1.586, 95% CI = 1.210–2.080, *P*_Z_ = 0.001) and recessive model (OR = 1.533, 95% CI = 1.273–1.846, *P*_Z_ = 0.000)] was significantly associated with COPD risk, consistent with findings in the overall analysis, indicating that some studies with *P*_HWE_ <0.05 did not affect the overall results, which were reliable.

**TABLE 5:** Meta-analysis results of the association between *GSTP1* rs1695 and COPD risk.

Genetic models	Ethnicity	Studies	Association test		M	Heterogeneity test		Publication bias	
			OR (95% CI)	*P*_Z_		I^2^ (%)	*P*_Q_	Egger’s test	Begg’s test
**GG vs. AA Homozygote model**	Overall	30	1.434 (1.054,1.952)	0.022	R	55.90	0.000	0.538	0.193
	Asian	21	1.151 (0.762,1.737)	0.504		59.70	0.000		
	Caucasian	9	2.064 (1.472,2.894)	0.000		11.00	0.343		
**GA vs. AA Heterozygote model**	Overall	30	0.966 (0.822,1.136)	0.678	R	58.00	0.000	0.529	0.986
	Asian	21	0.905 (0.742,1.104)	0.326		54.80	0.001		
	Caucasian	9	1.105 (0.820,1.490)	0.511		67.40	0.002		
**GG + GA vs. AA Dominant model**	Overall	30	1.027 (0.856,1.233)	0.771	R	71.00	0.000	0.732	0.929
	Asian	21	0.940 (0.747,1.183)	0.598		70.90	0.000		
	Caucasian	9	1.232 (0.901,1.686)	0.191		73.20	0.000		
**GG vs. GA + AA Recessive model**	Overall	31	1.395 (1.117,1.653)	0.000	F	45.40	0.004	0.556	0.255
	Asian	21	1.188 (0.960,1.496)	0.113		48.10	0.008		
	Caucasian	10	1.850 (1.393,2.457)	0.000		22.30	0.238		
**G vs. A Allele model**	Overall	31	1.061 (0.904,1.247)	0.496	R	75.90	0.000	0.759	0.507
	Asian	21	0.978 (0.790,1.211)	0.840		77.80	0.000		
	Caucasian	10	1.237 (0.974,1.570)	0.081		71.00	0.000		

Notes: M, model; F, fixed-effects model.

**FIGURE 5:**
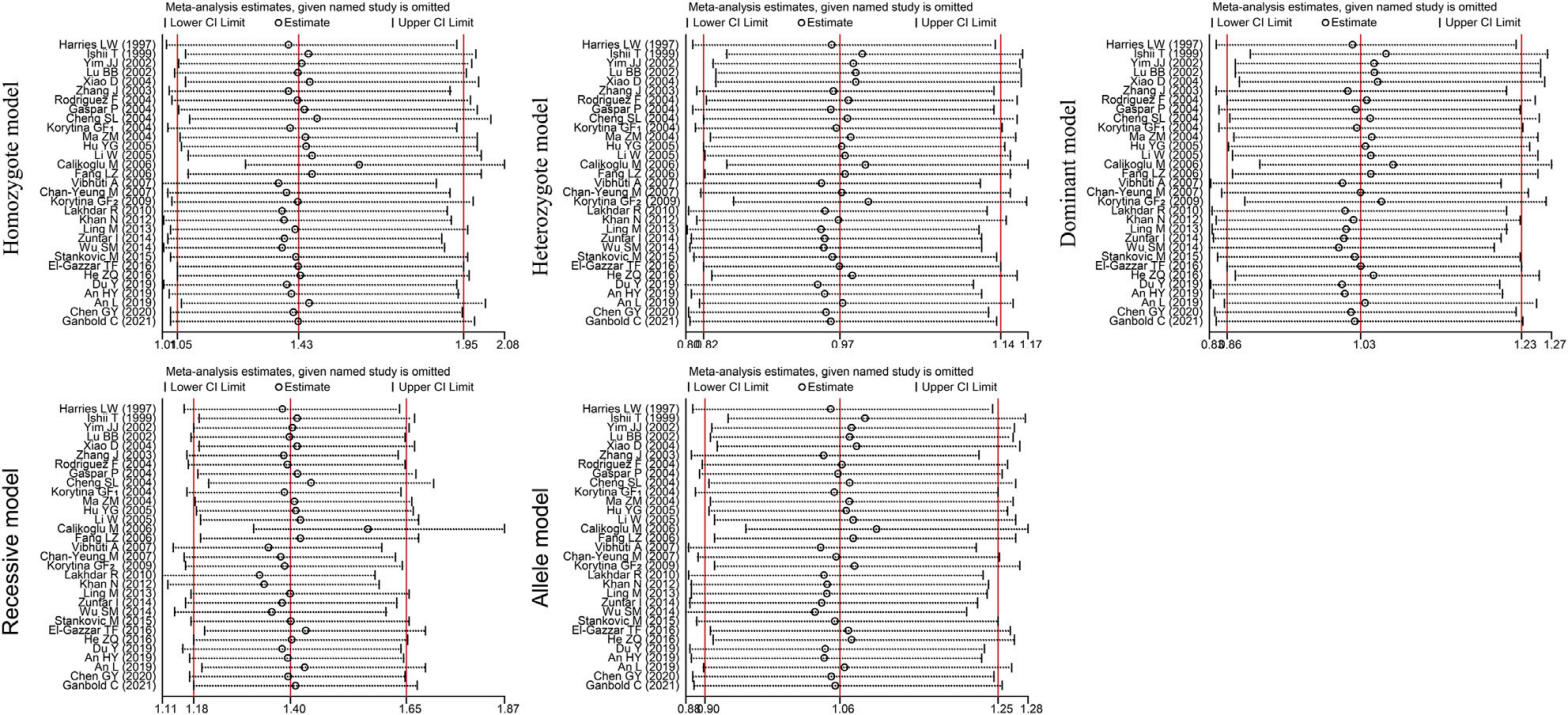
Sensitivity analysis diagram for *GSTP1* rs1695 polymorphism.

Egger’s and Begg’s tests did not reveal any publication bias (*P*_Egger_>0.05, *P*_Bgge_>0.05) among the included studies (*Table 5*). In addition, sensitivity analysis was performed by excluding the studies one by one to determine the impact of each study on heterogeneity (I^2^ >50%, P_Q_ <0.05). The OR values for all studies were within the 95% CI (Figure 5). These results indicate that the overall estimate was stable and the results were reliable.

### 3.6 Association between *GSTP1* rs1138272 polymorphism and COPD risk

The results of the meta-analysis of various models and subgroups used to explore the association between *GSTP1* rs1138272 and COPD risk are summarized in Table 6. In the overall analysis, significant heterogeneity was identified among the five genetic models (I^2^>50%, *P*_Q_<0.10). Therefore, the random-effects model was used to determine the pooled OR and its 95% CI. For the heterozygote model (OR = 1.908, *P*_Z_ = 0.013) and dominant model (OR = 1.81, *P*_Z_ = 0.018), *GSTP1* rs1138272 was significantly associated with COPD risk, indicating that the TC genotype is a risk factor for COPD. Subgroup analysis by ethnicity revealed a significant association of *GSTP1* rs1138272 with COPD risk among Caucasians for the homozygote model (OR = 2.570, *P*_Z_ = 0.112), heterozygote model (OR = 2.244, *P*_Z_ = 0.009) dominant model (OR = 2.303, *P*_Z_ = 0.009) and allele model (OR = 1.822, *P*_Z_ = 0.000). These results indicate that the TC genotype might be a risk factor for COPD among Caucasians. Since 2 studies had *P*_HWE_ <0.05 (Vibhuti et al., 2007; Korytina et al., 2009), we conducted a subgroup analysis of 5 studies with *P*_HWE_ ≥0.05 and observed that *GSTP1* rs1138272 was not significantly correlated with COPD risk [homozygote model (OR = 1.085, 95% CI = 0.768–7.656, *P*_Z_ = 0.918), heterozygote model (OR = 2.425, 95% CI = 0.768–7.656, *P*_Z_ = 0.131), dominant model (OR = 2.112, 95% CI = 0.743–6.006, *P*_Z_ = 0.161), recessive model (OR = 0.567, 95% CI = 0.158–2.038, *P*_Z_ = 0.385), and allele model (OR = 1.155, 95% CI = 0.742–1.797, *P*_Z_ = 0.524)], inconsistent with findings from the overall analysis.

**TABLE 6:** Meta-analysis results of the association between *GSTP1* rs1138272 and COPD risk.

Genetic models	Ethnicity	Studies	OR (95% CI)	*P*_Z_	M	Heterogeneity test		Publication bias	
						I^2^ (%)	*P*_Q_	Egger’s test	Begg’s test
**TT vs. CC Homozygote model**	Overall	5	1.284 (0.494,3.340)	0.608	R	74.60	0.003	0.639	1.000
	Asian	2	0.535 (0.194,1.471)	0.225		50.10	0.157		
	Caucasian	3	2.570 (0.803,8.230)	0.112		64.20	0.061		
**TC vs. CC Heterozygote model**	Over all	5	1.908 (1.144,3.184)	0.013	R	77.10	0.002	0.112	0.086
	Asian	2	1.528 (0.518,4.509)	0.442		88.40	0.003		
	Caucasian	3	2.244 (1.223,4.117)	0.009		63.00	0.067		
**TT + TC vs. CC Dominant model**	Overall	5	1.810 (1.105,2.966)	0.018	R	77.60	0.001	0.138	0.221
	Asian	2	1.327 (0.559,3.148)	0.521		82.80	0.016		
	Caucasian	3	2.303 (1.236,4.290)	0.009		68.6	0.041		
**TT vs. TC + CC Recessive model**	Overall	5	0.844 (0.350,2.036)	0.706	R	75.60	0.003	0.655	0.806
	Asian	2	0.344 (0.053,2.254)	0.266		86.30	0.007		
	Caucasian	3	1.559 (0.792,3.070)	0.199		23.30	0.272		
**T vs. C Allele model**	Overall	5	1.375 (0.958,1.974)	0.084	R	78.60	0.001	0.161	0.221

Notes: M, model; F, fixed-effects model.

**FIGURE 6:**
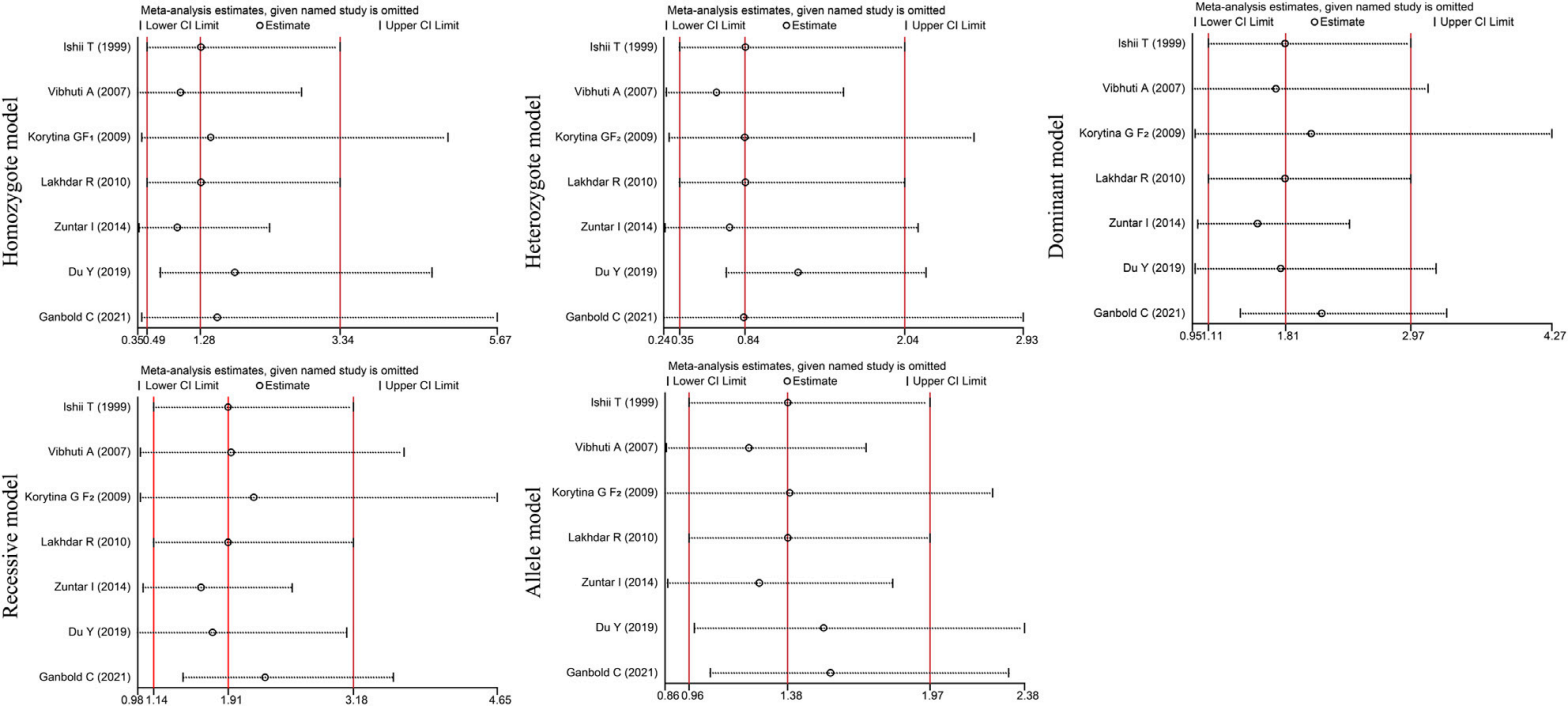
Sensitivity analysis diagram of the *GSTP1* rs1138272 polymorphism

Egger’s and Begg’s tests did not reveal any publication bias among the included studies (*P*_Egger_>0.05, *P*_Bgge_>0.05) (*Table 6*). Given the significant heterogeneity (I^2^>50%, *P*_Q_<0.05) and inconsistent results caused by articles with *P*_HWE_<0.05, we performed a sensitivity analysis by excluding the studies one by one. The OR values of all studies were within the 95% CI (Figure 6). These results indicate that the overall estimate was stable and the results were reliable.

## 4 Discussion

Chronic obstructive pulmonary disease (COPD) is a polygenic disease that is caused by various environmental factors and genetic factors (Silverman, 2020). The genes implicated in COPD occurrence are those that participate in anti-proteolysis, metabolism of toxic cigarette substances, airway hyperresponsiveness, inflammatory responses to smoking, and oxidative stress (Bossé, 2012). Among them, oxidative stress contributes to the upregulation of proinflammatory cytokine genes expression, enhances inflammatory responses, and damages the airway epithelium as well as the pulmonary interstitium and is a key driver of COPD development (Wu et al., 2014; Zhang et al., 2015). *EPHX1* and *GSTP1* are key oxidation-inhibiting enzymes which are xenobiotic metabolism enzyme genes. They are mainly involved in the first metabolism of foreign xenobiotic substances in the lungs, including cigarette smoke, oxides, and intermediate products of reactive oxides (An et al., 2019a). Although they have been extensively studied, the relationship between their mutations and the pathogenesis as well as risk of COPD is controversial. Thus, there is a need to explore the roles of *EPHX1* and *GSTP1* gene polymorphisms in COPD development to inform the development of diagnostic and therapeutic strategies for the disease. This meta-analysis investigated the association of *EPHX1* (rs1051740 and rs2234922) and *GSTP1* (rs1695 and rs1138272) polymorphisms with the risk of COPD. Since the ethnic background of gene-gene and gene-environment interactions affect SNP and disease risk, we conducted subgroup analysis based on ethnicity to determine the association between *EPHX1* and *GSTP1* gene polymorphisms and COPD risk in different ethnic groups.

Our meta-analysis of *EPHX1* rs1051740 showed that the C allele of *EPHX1* rs1051740 may be a risk factor for COPD. It was reported that a T/C mutation on codon 113 of exon 3 of *EPHX1*, substituting Tyr113 with His113, reduces the enzymatic activities of *EPHX1* by 40%–50%, which increases the oxidation rate beyond the antioxidative capacity, resulting in the accumulation of reactive oxygen species in the organism and eventually, cell and lung tissue damage (Hassett et al., 1994). Clinical studies have demonstrated that the C allele is associated with lung dysfunction, reduced glutathione levels, and elevated malondialdehyde (MDA) levels in COPD patients (Vibhuti et al., 2007). In the subgroup analysis of *EPHX1* rs1051740, it was found that the C allele is a risk factor for COPD in Asians. Furthermore, compared with genotype TT, genotype CC is a risk factor for COPD in Caucasians. These results are consistent with the observations that *EPHX1* rs1051740 mutations decrease *EPHX1* enzyme activity leading to an increase in the risk of COPD. A previous meta-analysis of 19 studies published in 2016 by An L et al. did not find any association between *EPHX1* rs1051740 and COPD risk in Asians (OR = 0.92, *P*_Z=_0.31) and Caucasians (OR = 1.01, *P*_Z=_0.65) (An et al., 2016b). However, this study only used the allele model to pool analysis, which has greater limitations and needs further in-depth mining and research. Differences among the meta-analyses may also be attributed to the number of included studies that used the allelic model to perform the meta-analysis and publication bias. Here, we included all available studies that met our inclusion criteria (28 studies) to study the association between different genotypes of *EPHX1* and COPD risk and used Egger’s and Begg’s tests to rule out any publication bias. And we also used the trim-and-fill method and sensitivity analysis to enhance the study’s rigor and reliability.

Furthermore, when a G/A mutation on codon 139 of *EPHX1* exon 4 replaces Hisl39 with Arg139, it increases the activities of *EPHX1* by 25% (Luo et al., 2019). Our meta-analysis findings indicate that the G allele of the *EPHX1* rs2234922 gene may confer protection against COPD. Furthermore, in the subgroup analysis, we observed that Asians with the GA genotype had a decreased risk of COPD compared to individuals with the AA genotype. However, in the case of Caucasians, we did not identify any statistically significant associations. In contrast, studies of An L found that *EPHX1* rs2234922 is not associated with COPD pathogenesis (OR = 1.01, *P*_Z_ = 0.65) (An et al., 2016b). Lee J et al. concluded that although a statistically significant correlation was not observed, the presence of *EPHX1* rs2234922 correlated with reduced COPD risk, consistent with the theory that the GA genotype of *EPHX1* can increase the detoxification ability of the *EPHX1* enzyme (Lee et al., 2011). We postulated that (rs1051740 and rs2234922) polymorphisms affect its enzymatic activities, which further involve the oxidative/antioxidative balance. Further investigations are required to establish if the polymorphisms of *EPHX1* (rs1051740 and rs2234922) are significantly associated with COPD risk.

The tightly linked gene-gene interactions were observed between *EPHX1* and *GSTP1* gene polymorphisms, and alterations in the combined *EPHX1*-*GSTP1* detoxification activity may affect COPD development (Ganbold et al., 2021). In view of the relationship between *GSTP1* and *EPHX1*, we simultaneously investigated the correlation between *GSTP1* polymorphisms and COPD risk. It was found that the GG genotype on *GSTP1* rs1695 may be a risk factor for COPD. Subgroup analysis showed that Caucasians with the GG genotype were more likely to develop COPD than those with GA or AA genotypes, but the GG genotype was not associated with an increased risk of COPD among Asians. These findings are consistent with those of a previous meta-analysis published in 2010 that *GSTP1* rs1695 is associated with increased COPD risk among Caucasians in the recessive model (OR = 1.59, *P*_Z=_0.001) but not among Asians (OR = 0.93, *P*_Z_ = 0.64) (Zhong et al., 2010). However, another meta-analysis of 17 studies published in 2015 by Yang L found that there is no significant correlation between *GSTP1* rs1695 polymorphism and COPD risk in any genetic model (Yang et al., 2015). The association between *GSTP1* rs1695 polymorphism and COPD risk remains controversial, and no meta-analysis update has been conducted recently. Thus, we conducted a meta-analysis. In our study, we included 31 articles, including 5 newly published studies in the recent 7 years, and the analysis was more comprehensive and rigorous. Our results are more reliable than those of the other meta-analyses. It has been shown that the A/G mutations on *GSTP1*’s exon-5, which replace Ile105 with Val105, result in changing the volume and hydrophobicity of amino acids and inhibiting the enzyme’s activities as well as thermal stability, thereby reducing its detoxification capacity (Watson et al., 1998). It results in excess amounts of oxidants and free radicals in lung tissues and promotes airway tissue inflammation, which can cause bronchitis, emphysema, and COPD (Ganbold et al., 2021). This is consistent with our finding that the *GSTP1* rs1695 GG genotype increases COPD risk. Large-sample clinical research showed that *GSTP1* rs1695 was related to the rapid decline of lung function, which indirectly supported this evidence (He et al., 2004).

Our meta-analysis of *GSTP1* rs1138272 showed that the TC genotype is a risk factor for COPD, and subgroup analysis showed that Caucasians carrying the TC genotype are more likely to suffer from COPD. It has been shown that the frequency of *GSTP1* rs1138272 TC genotype in COPD patients was significantly higher than in normal people (28.57% vs. 14.45%), indicating that the *GSTP1* rs1138272 polymorphism may be associated with COPD risk (Korytina et al., 2009). In contrast, Ganbold C et al. (Ganbold et al., 2021) concluded that *GSTP1* rs1138272 polymorphisms are not correlated with COPD risk (OR = 1.38, *p* = 0.381). It is reported that the T/C variant of *GSTP1* on exon 6 replaces Ala114 with Val114 without changing enzymatic activities (Watson et al., 1998). However, which is inconsistent with our findings. Our conclusion should be further validated because the number of studies involving the rs1138272 polymorphism of *GSTP1* and the risk for COPD is small. We found that *GSTP1* (rs1695 and rs1138272) mutations are associated with an increased risk of COPD. Given the correlation between GSTP and *EPHX1*, further studies should be performed to establish if *EPHX1*-*GSTP1* interactions influence COPD development.

The *P*_HWE_<0.05 of the control group in the original article indicates that there may be a potential deviation in the study during control selection or genotyping errors (Lee et al., 2014). To avoid such deviations, we conducted subgroup analysis on studies with *P*_HWE_≥0.05 and found that only subgroup analysis results from *GSTP1* rs1138272 differed from the original results. Our analysis found that data from 2 of the 5 studies involving *GSTP1* rs1138272 (*P*_HWE_≥0.05) could not be calculated using the factor model and another 3 studies had a high heterogeneity (I^2^ >50%, *P*_Q_<0.05), indicating that the deviation may be caused by the high heterogeneity between studies and the small number of studies. Sensitivity analysis showed that OR values for all of the studies were within the 95% CI, indicating that the results were stable and reliable. After subgroup analysis according to *P*_HWE_≥0.05, the heterogeneity of *EPHX1* 1051740 (heterozygote model, dominant model and recessive model), *EPHX1* rs2234922 (heterozygote model, dominant model and allele model), and *GSTP1* rs1695 (homozygote model, heterozygote model, dominant model, recessive model and allele model) were decreased, so whether the control group included in the study conformed to *P*_HWE_ ≥0.05 may be one of the sources of partial result heterogeneity.

## 5 Strengths and limitations of the study

The key strengths of this study are as follows. Firstly, strict inclusion and exclusion criteria were used to comprehensively assess the association of the polymorphisms of *EPHX1* (rs1051740 and rs2234922) and *GSTP1* (rs1695 and rs1138272) with the risk of COPD. Moreover, subgroup analysis was performed on different ethnicities to determine the effects of these polymorphisms on COPD susceptibility in diverse populations. However, this study has some limitations. For instance, our results are based on individual unadjusted estimates, and therefore, a more accurate prediction model need to be established after adjusting for potential confounding factors, such as sex, age, body mass index, lung functions, smoking status, and other environmental factors. However, subgroup analysis did not reveal whether these factors are associated with gene polymorphisms. Secondly, the results obtained from the subgroup analyses may be limited by the small number of studies involving African populations. Thirdly, although genetic and environmental factors may increase COPD risk, gene-gene and gene-environment interactions could not be assessed because of the limited data available. Finally, some of the studies included in this meta-analysis had significant heterogeneity which decreases the reliability of the final results.

## 6 Conclusion

*EPHX1* (rs1051740 and rs2234922) and *GSTP1* (rs1695 and rs1138272) polymorphisms are associated with the risk of COPD. The C allele of *EPHX1* rs1051740 may increase the risk of COPD, especially among Asians, whereas the CC genotype may be a risk factor for COPD among Caucasians. In contrast, the G allele of *EPHX1* rs2234922 may protect against COPD, especially the GA genotype significantly reducing COPD risk in Asians. The G allele of *GSTP1* rs1695 may increase COPD risk, especially among Africans, whereas the TC genotype of *GSTP1* rs1138272 may increase COPD risk, especially among Caucasians. These results indicate that *EPHX1* and *GSTP1* gene polymorphisms play key roles in COPD pathogenesis. Therefore, they are potential diagnostic and therapeutic targets in COPD. However, our conclusions should be validated in larger studies. Moreover, further analysis of gene-gene and gene-environment interactions should be performed to elucidate the mechanisms of COPD pathogenesis.

## Data Availability

The original contributions presented in the study are included in the article/Supplementary Material, further inquiries can be directed to the corresponding author.
