# Supplementary material for: EPHX1 and GSTP1 polymorphisms are associated with COPD risk: a systematic review and meta-analysis
Source: Front Genet. 2023 May 22;14:1128985. doi: 10.3389/fgene.2023.1128985 (PMC10239837; doi:10.3389/fgene.2023.1128985)
Supplement: Supplementary file 1 [file Table1.DOCX]

**Association between polymorphisms of EPHX1 rs1051740, rs2234922 and GSTP1 rs1695, rs1138272 with COPD correlation:a systematic review and meta-analysis**

**Supplement: content**

| **TABLE S1a. Search strategy in Pubmed** ([https://pubmed. ncbi. nlm. nih. gov/](https://pubmed.ncbi.nlm.nih.gov/)) | | | |
| --- | --- | --- | --- |
| **COPD** | 1. Pulmonary Disease, Chronic Obstructive[MeSH Terms]  2. Chronic Obstructive Pulmonary Diseases[MeSH Terms]  3. Chronic Obstructive Lung Disease[MeSH Terms]  4. COAD[Title/Abstract]  5. COPD[Title/Abstract]  6. Chronic Obstructive Airway Disease[MeSH Terms]  7. Chronic Obstructive Pulmonary Disease[MeSH Terms]  8. Airflow Obstruction, Chronic[MeSH Terms]  9. Airflow Obstructions, Chronic[MeSH Terms]  10. Chronic Airflow Obstructions[MeSH Terms]  11. Chronic Airflow Obstruction[MeSH Terms]  12. 1 OR 2 OR 3 OR 4 OR 5 OR 6 OR 7 OR 8 OR 9 OR 10 OR 11 | | |
| **Gene** | 13. Gene*[MeSH Terms]  14. Cistron*[Title/Abstract]  15. Genetic Material*[Title/Abstract]  16. Material, Genetic[MeSH Terms]  17. Materials, Genetic[MeSH Terms]  18. 13 OR 14 OR 15 OR 16 OR 17 | | |
| **Genetic Polymorphism** | 19. Polymorphism, Genetic[MeSH Terms]  20. Genetic Polymorphism*[Title/Abstract]  21. Gene Polymorphisms*[Title/Abstract]  22. Polymorphism, Gene[MeSH Terms]  23. Polymorphisms, Gene[MeSH Terms]  24. Polymorphism (Genetics)  25. Polymorphisms (Genetics)  26. 19 OR 20 OR 21 OR 22 OR 23 OR 24 OR 25 | | |
| **Genetic Variation** | 27. Genetic Variation*[MeSH Terms]  28. Variations, Genetic[MeSH Terms]  29. Variation, Genetic[MeSH Terms]  30. Diversity, Genetic[MeSH Terms]  31. Diversities, Genetic[MeSH Terms]  32. Genetic Diversit*[Title/Abstract]  33. 27 OR 28 OR 29 OR 30 OR 31 OR 32 | | |
| **SNP** | 34. Polymorphism, Single Nucleotide[MeSH Terms]  35. Nucleotide Polymorphism, Single[MeSH Terms]  36. Nucleotide Polymorphisms, Single[MeSH Terms]  37. Polymorphisms, Single Nucleotide[MeSH Terms]  38. Single Nucleotide Polymorphism*[Title/Abstract]  39. SNPs[Title/Abstract]  40. 34 OR 35 OR 36 OR 37 OR 38 OR 39 | | |
| **Combine** | 41. 18 OR 26 OR 33 OR 40 | | |
| **EPHX1** | 42. "EPHX1 protein, human" [Supplementary Concept]  43. EPHX1[Title/Abstract]  44. epoxide hydrolase 1, microsomal (xenobiotic), human  45. mEPHX[Title/Abstract]  46. mEH[Title/Abstract]  47. mEPH[Title/Abstract]  48. EH[Title/Abstract]  49. 42 OR 43 OR 44 OR 45 OR 46 OR 47 OR 48 | **GSTP1** | 50. Glutathione S-Transferase pi[MeSH Terms]  51. Glutathione S-Transferase pi[Title/Abstract]  52. Glutathione S Transferase pi[MeSH Terms]  53. Glutathione S Transferase pi[Title/Abstract]  54. GSTP1[Title/Abstract]  55. GST Class-phi[MeSH Terms]  56. GST Class-phi[Title/Abstract]  57. GST Class-phi[MeSH Terms]  58. GST Class-phi[Title/Abstract]  59. Class-phi, GST[MeSH Terms]  60. GST Class phi[MeSH Terms]  61. GST Class phi[Title/Abstract]  62. Glutathione Transferase P1-1[MeSH Terms]  63. Glutathione Transferase P1-1[Title/Abstract]  64. Glutathione Transferase P1 1[MeSH Terms]  65. Glutathione Transferase P1 1[Title/Abstract]  66. Transferase P1-1, Glutathione[MeSH Terms]  67. GSTP1 Glutathione D-Transferase[MeSH Terms]  68. D-Transferase, GSTP1 Glutathione[MeSH Terms]  69. GSTP1 Glutathione D Transferase[MeSH Terms]  70. Glutathione D-Transferase, GSTP1[MeSH Terms]  71. 50 OR 51 OR 52 OR 53 OR 54 OR 55 OR 56 OR 57 OR 58 OR 59 OR 60 OR 61 OR 62 OR 63 OR 64 OR 65 OR 66 OR 67 OR 68 OR 69 OR 70 |
| **Combine** | EPHX1: 12 AND 41 AND 49 | GSTP1: 12 AND 41 AND 71 | |

| **TABLE S1b. Search strategy in EMBASE (**<https://www.embase.com/>**)** | | | |
| --- | --- | --- | --- |
| **COPD** | #1 'chronic obstructive lung disease'/exp  #2 'chronic obstructive pulmonary diseases':ti,ab,kw  #3 'chronic obstructive lung disease':ti,ab,kw  #4 'coad':ti,ab,kw  #5 'pulmonary disease, chronic obstructive'  #6 'copd':ti,ab,kw  #7 'chronic obstructive airway disease':ti,ab,kw  #8 'chronic obstructive pulmonary disease':ti,ab,kw  #9 'airflow obstruction, chronic'  #10 'airflow obstructions, chronic'  #11 'chronic airflow obstructions':ti,ab,kw  #12 'chronic airflow obstruction':ti,ab,kw  #13 #1 OR #2 OR #3 OR #4 OR #5 OR #6 OR #7 OR #8 OR #9 OR #10 OR #11 OR #12 | | |
| **Gene** | #14 'gene'/exp  #15 'gene':ti,ab,kw  #16 'cistron':ti,ab,kw  #17 'genetic material':ti,ab,kw  #18 'material, genetic'  #19 'materials, genetic'  #20 14 OR 15 OR 16 OR 17 OR 18 OR 19 | | |
| **Genetic Polymorphism** | #21 'genetic polymorphism'/exp  #22 polymorphism:ti,ab,kw  #23 'polymorphism, gene'  #24 'polymorphism, genetic'  #25 #21 OR #22 OR #23 OR #24 | | |
| **Genetic Variation** | #26 'genetic variation'/exp  #27 'genetic variability'/exp  #28 'diversity, genetic'  #29 'variation, genetic'  #30 #26 OR #27 OR #29 | | |
| **SNP** | #31 'single nucleotide polymorphism'/exp  #32 snps:ti,ab,kw  #33 'polymorphism, single nucleotide'  #34 'nucleotide polymorphism, single'  #35 #31 OR #32 OR #33 OR #34 | | |
|  | #36 #20 OR #25 OR #30 OR #35 | | |
| **EPHX1** | #37 mephx:ab,ti  #38 meh:ab,ti  #39 meph:ab,ti  #40 eh:ab,ti  #41 'ephx1 gene'/exp  #42 'ephx1 protein'/exp  #43 'ephx1 protein human'/exp  #44 #37 OR #38 OR #39 OR #40 OR #41 OR #42 OR #43 | **GSTP1** | #45. 'glutathione transferase P1'/exp  #46. 'gstp1 gene'/exp  #47. 'gstp1 protein human'/exp  #48. gstp1:ab,ti  #49. #45 OR #46 OR #47 OR #48 |
| **Combine** | EPHX1: #13 AND #36 AND #44 | GSTP1: #13 AND #36 AND #49 | |

| **TABLE S1c. Search strategy in Web Of Science(**<https://www.webofscience.com/>**)** | | | |
| --- | --- | --- | --- |
| **COPD** | 1. TS=(copd)  2. TS=(Chronic Obstructive Pulmonary Diseases)  3. TS=(Chronic Obstructive Lung Disease)  4. TI=(COAD)  5. TI=(COPD)  6. TS=(Chronic Obstructive Airway Disease)  7. TS=(Chronic Obstructive Pulmonary Disease)  8. TS=(Chronic Airflow Obstruction)  9. 1 OR 2 OR 3 OR 4 OR 5 OR 6 OR 7 OR 8 | | |
| **Gene** | 10. TS=(Gene*)  11. TI=(Cistron*)  12. TI=(Genetic Material)  13. 10 OR 11 OR 12 | | |
| **Genetic Polymorphism** | 14. TS=(Polymorphism, Genetic)  15. TI=(Genetic Polymorphism*)  16. TI=(Gene Polymorphisms*)  17. TS=(Polymorphism, Gene)  18. TS=(Polymorphism (Genetics)  19. 14 OR 15 OR 16 OR 17 OR 18 | | |
| **Genetic Variation** | 20. TS=(Genetic Variation*)  21. TS=(Variation, Genetic)  22. TS=(Diversity, Genetic)  23. TI=(Genetic Diversit*)  24. 20 OR 21 OR 22 OR 23 | | |
| **SNP** | 25. TS=(Polymorphism, Single Nucleotide)  26. TS=(Nucleotide Polymorphism, Single)  27. TS=(Polymorphisms, Single Nucleotide)  28. TS=(Single Nucleotide Polymorphism*)  29. TI=(Single Nucleotide Polymorphism*)  30. TI=(SNPs)  31. TS=(SNPs)  32. 25 OR 26 OR 27 OR 28 OR 29 OR 30 OR 31 | | |
| **Combine** | 36. 20 OR 25 OR 30 OR 35 | | |
| **EPHX1** | 37. TS=(EPHX1)  38. TI=(EPHX1)  39. TS=(epoxide hydrolase 1, microsomal (xenobiotic), human)  40. TS=(ephx1 gene)  41. TI=(ephx1 gene)  42. TI=(ephx1 protein)  43. TS=(mephx)  44. TI=(mephx)  45. TI=(meh)  46. TS=(meh)  47. TS=(mEPH)  48. TI=(mEPH)  49. AB=(mEPH)  50. TS=(EH)  51. TI=(EH)  52. AB=(EH)  53. 37 OR 38 OR 39 OR 40 OR 41 OR 42 OR 43 OR 44 OR 45 OR 46 OR 47 OR 48 OR 49 OR 50 OR 51 OR 52 | **GSTP1** | 53. TS=(glutathione transferase P1)  54 TI=(glutathione transferase P1).  55. AB=(glutathione transferase P1)  56. AB=(gstp1 gene)  57. TS=(gstp1 gene)  58. TI=(gstp1 gene)  59. TS=(gstp1 protein human)  60. TI=(gstp1 protein human)  61. AB=(gstp1 protein human)  62. TS=( gstp1)  63. TI=( gstp1)  64. AB=( gstp1)  65. 53 OR 54 OR 55 OR 56 OR 57 OR 58 OR 59 OR 60 OR 61 OR 62 OR 63 OR 64 |
| **Combine** | EPHX1: 9 AND 36 AND 53 | GSTP1: 9 AND 36 AND 65 | |

| **TABLE S1d. Search strategy in CENTRAL(**<https://www.proquest.com/>**)** | | | |
| --- | --- | --- | --- |
| **COPD** | S1. noft(chronic obstructive pulmonary diseases)  S2. mainsubject(pulmonary disease, chronic obstructive)  S3. mainsubject(chronic obstructive lung disease)  S4. ti(COAD)  S5. ti(COPD)  S6. noft(chronic obstructive airways disease)  S7. mainsubject(airflow obstruction, chronic)  S8. mainsubject(airflow obstruction, chronic)  S9. mainsubject(chronic airflow obstruction*)  S10. S1 OR S2 OR S3 OR S4 OR S5 OR S6 OR S7 OR S8 OR S9 | | |
| **Gene** | S11. mainsubject(gene*)  S12. noft(gene*)  S13. noft(cistron*)  S14. noft(genetic material*)  S15. noft(material, genetic)  S17. noft(materials, genetic)  S18. S11 OR S12 OR S13 OR S14 OR S15 OR S16 OR S17 | | |
| **Genetic Polymorphism** | S19. noft(polymorphism, genetic)  S20. mainsubject(genetic polymorphism*)  S21. mainsubject(gene polymorphisms*)  S22. noft(polymorphism, genetic)  S23. noft(polymorphisms, gene)  S24. noft(genetics of polymorphism and variation)  S25. noft(polymorphisms (genetics)  S26. S19 OR S20 OR S21 OR S22 OR S23 OR S24 OR S25 | | |
| **Geneti Variation** | S26. mainsubject(genetic variation*)  S27. noft(variations, genetic)  S28. noft(variation, genetic)  S29. noft(diversity, genetic)  S30. noft(diversities, genetic)  S31. noft(genetic diversit*)  S32. S26 OR S27 OR S28 OR S29 OR S30 OR S31 | | |
| **SNP** | S33. mainsubject(single nucleotide polymorphism*)  S34. ti(snps)  S35. noft(polymorphisms, single nucleotide)  S36. noft(nucleotide polymorphisms, single)  S37. noft(nucleotide polymorphism, single)  S38. noft(polymorphism, single nucleotide)  S39. S33 OR S34 OR S35 OR S36 OR S37 OR S38 | | |
| **Combine** | S40. S18 OR S26 OR S38 OR S39 | | |
| **EPHX1** | S40. mainsubject(EPHX1)  S41. noft(EPHX1)  S42. mainsubject(microsomal epoxide hydrolase)  S43. mainsubject(epoxide hydrolase)  S44. noft(meh)  S45. noft(mephx)  S46. noft(mEPH)  S47. noft(EH)  S48. S40 OR S41 OR S42 OR S43 OR S44 OR S45 OR S46 OR S47 | GSTP1 | 49. mainsubject(glutathione transferase P1)  50. noft(glutathione transferase P1)  51. noft(gstp1 gene)  52. noft(gstp1 protein human)  53. noft(gstp1)  54. 49 OR 50 OR 51 OR 52 OR 53 |
| **Combine** | EPHX1: S10 AND S40 AND S48 | GSTP1: S10 AND S40 AND S54 | |

| **TABLE S1e. Search strategy in SCOUPS(**<https://www.scopus.com/>**)** | | | |
| --- | --- | --- | --- |
| **COPD** | 1. TITLE-ABS-KEY ( chronic AND obstructive AND pulmonary AND disease* )  2. TITLE-ABS-KEY ( chronic AND obstructive AND lung AND disease* )  3. TITLE-ABS-KEY ( coad )  4. TITLE-ABS-KEY ( copd )  5. TITLE-ABS-KEY ( chronic AND obstructive AND airway AND disease* )  6. TITLE-ABS-KEY ( chronic AND airflow AND obstruction )  7. 1 OR 2 OR 3 OR 4 OR 5 OR 6 | | |
| **Gene** | 8. TITLE-ABS-KEY ( gene* )  9. TITLE-ABS-KEY ( cistron* )  10. TITLE-ABS-KEY ( genetic AND material* )  11. 8 OR 9 OR 10 | | |
| **Genetic Polymorphism** | 12. TITLE-ABS-KEY ( genetic AND polymorphism* )  13. TITLE-ABS-KEY ( gene AND polymorphisms* )  14. 12 OR 13 | | |
| **Genetic Variation** | 15. TITLE-ABS-KEY ( genetic AND variation* )  16. TITLE-ABS-KEY ( genetic AND diversit* )  17. 15 OR 16 | | |
| **SNP** | 18. TITLE-ABS-KEY ( single AND nucleotide AND polymorphism )  19. TITLE-ABS-KEY ( snps )  20. 18 OR 19 | | |
| **Combine** | 21. 11 OR 14 OR 17 OR 20 | | |
| **EPHX1** | 22. TITLE-ABS-KEY ( ephx1 )  23. TITLE-ABS-KEY ( mephx )  24. TITLE-ABS-KEY ( meh )  25. TITLE-ABS-KEY ( meph )  26. TITLE-ABS-KEY ( eh )  27. TITLE-ABS-KEY ( ephx1 AND gene )  28. TITLE-ABS-KEY ( ephx1 AND protein )  29. 22 OR 23 OR 24 OR 25 OR 26 OR 27 OR 28 | **GSTP1** | 30. TITLE-ABS-KEY ( glutathione AND transferase AND p1  31. TITLE-ABS-KEY ( gstp1 AND gene )  32. TITLE-ABS-KEY ( gstp1 AND protein AND human )  33. TITLE-ABS-KEY ( gstp1 )  34. 30 OR 31 OR 32 OR 33 |
| **Combine** | EPHX1: 7 AND 21 AND 29 | GSTP1: 7 AND 21 AND 34 | |

| **TABLE S1f. Search strategy in Cochrane(**<https://www.cochrane.org/>**)** | | | |
| --- | --- | --- | --- |
| **COPD** | 1. MeSH descriptor: [Pulmonary Disease, Chronic Obstructive] explode all trees | | |
| **EPHX1** | 2. (mephx):ti,ab,kw  3. (meh):ti,ab,kw  4. (ephx1):ti,ab,kw  5. (mEH):ti,ab,kw  6. (mEPH):ti,ab,kw  7. (EH):ti,ab,kw  8. 2 OR 3 OR 4 OR 5 OR 6 OR 7 | **GSTP1** | 9. MeSH descriptor: [Glutathione S-Transferase pi] explode all trees  10. (GSTP1):ti,ab,kw  11. (gstp1 gene):ti,ab,kw  12. 9 OR 10 OR 11 |
| **Combine** | EPHX1: 1 AND 8 | GSTP1: 1 AND 12 | |

| **TABLE S1g. Search strategy in CINAHL(**<https://web.s.ebscohost.com/>**)** | | | |
| --- | --- | --- | --- |
| **COPD** | S1. SU chronic obstructive pulmonary disease  S2. SU pulmonary disease, chronic obstructive  S3. SU chronic obstructive lung disease  S4. TI COAD  S5. AB COAD  S6. TI COPD  S7. AB COPD  S8. SU chronic obstructive airway disease  S9. TI airflow obstruction, chronic  S10. AB airflow obstruction, chronic  S11. TI chronic airflow obstruction  S12. AB chronic airflow obstruction  S13. S1 OR S2 OR S3 OR S4 OR S5 OR S6 OR S7 OR S8 OR S9 OR S10 OR S11 OR S12 | | |
| **Gene** | S14. SU gene*  S15. TI cistron*  S16. AB cistron*  S17. TI genetic material*  S18. AB genetic material*  S19. TI material, genetic  S20. AB material, genetic  S21. S14 OR S15 OR S16 OR S17 OR S18 OR S19 OR S20 | | |
| **Genetic Polymorphism** | S22. SU genetic polymorphism*  S23. SU gene polymorphism*  S24. TI polymorphism, gene  S25. AB polymorphism, gene  S26. TI polymorphism, genetic  S27. AB polymorphism, genetic  S28. S22 OR S23 OR S24 OR S25 OR S26 OR S27 | | |
| **Genetic Variation** | S29. SU genetic variation*  S30. TI variation, genetic  S31. AB variation, genetic  S32. TI diversity, genetic  S33. AB diversity, genetic  S34. SU genetic diversity*  S35. S29 OR S30 OR S31 OR S32 OR S33 OR S34 | | |
| **SNP** | S36. SU snps  S37. SU ( snps: impact on gene function and phenotype )  S38. TI polymorphism, single nucleotide  S39. AB polymorphism, single nucleotide  S40. TI nucleotide polymorphism, single  S41. AB nucleotide polymorphism, single  S42. SU single nucleotide polymorphism*  S43. S36 OR S37 OR S38 OR S39 OR S40 OR S41 OR S42 | | |
| **Combine** | S44. S21 OR S28 OR S35 OR S43 | | |
| **EPHX1** | S45. SU EPHX1  S46. TI meh  S47. AB meh  S48. TI mephx  S49. AB mephx  S50. TI meph  S51. AB mEPH  S52. TI EH  S53. AB EH  S54. S45 OR S46 OR S47 OR S48 OR S49 OR S50 OR S51 OR S52 OR S53 | **GSTP1** | S55. AB glutathione transferase P1  S56. SU glutathione transferase P1  S57. gstp1 gene  S58. AB gstp1 gene  S59. AB gstp1 protein human  S60. SU gstp1 protein human  S61. SU gstp1  S62. TI gstp1  S63. AB gstp1  S64. S55 OR S56 OR S57 OR S58 OR S59 OR S60 OR S61 OR S62 OR S63 |
| **Combine** | EPHX1: S13 AND S44 AND S54 | GSTP1: S13 AND S44 AND S64 | |
